# Supplementary material for: Akebia Saponin D Inhibits the Inflammatory Reaction by Inhibiting the IL-6-STAT3-DNMT3b Axis and Activating the Nrf2 Pathway
Source: Molecules. 2022 Sep 22;27(19):6236. doi: 10.3390/molecules27196236 (PMC9614599; doi:10.3390/molecules27196236)
Supplement: Supplementary file 1 [file molecules-27-06236-s001.zip › Editing Certificate P4SYWN7H_2A9B-63DA-0775-E741-3DF8.pdf]

Editing Certificate

This document certifies that the manuscript

Akebia Saponin D inhibits inflammatory reaction through in-hibiting IL-6-STAT3-DNMT3b axis and activating Nrf2 path-way

prepared by the authors

Jin-Fang Luo, Chon-Kit Lio and Hua Zhou

was edited for proper English language, grammar, punctuation, spelling, and overall style by one or more of the highly qualified native English speaking editors at AJE.

This certificate was issued on **August 16, 2022** and may be verified on the AJE website using the verification code **2A9B-63DA-0775-E741-3DF8**.

Neither the research content nor the authors' intentions were altered in any way during the editing process. Documents receiving this certification should be English-ready for publication; however, the author has the ability to accept or reject our suggestions and changes. To verify the final AJE edited version, please visit our verification page at aje.com/certificate. If you have any questions or concerns about this edited document, please contact AJE at support@aje.com.

AJE provides a range of editing, translation, and manuscript services for researchers and publishers around the world.
For more information about our company, services, and partner discounts, please visit aje.com.
