# Supplementary figures and images for: Akebia Saponin D Inhibits the Inflammatory Reaction by Inhibiting the IL-6-STAT3-DNMT3b Axis and Activating the Nrf2 Pathway
Source: Molecules. 2022 Sep 22;27(19):6236. doi: 10.3390/molecules27196236 (PMC9614599; doi:10.3390/molecules27196236)

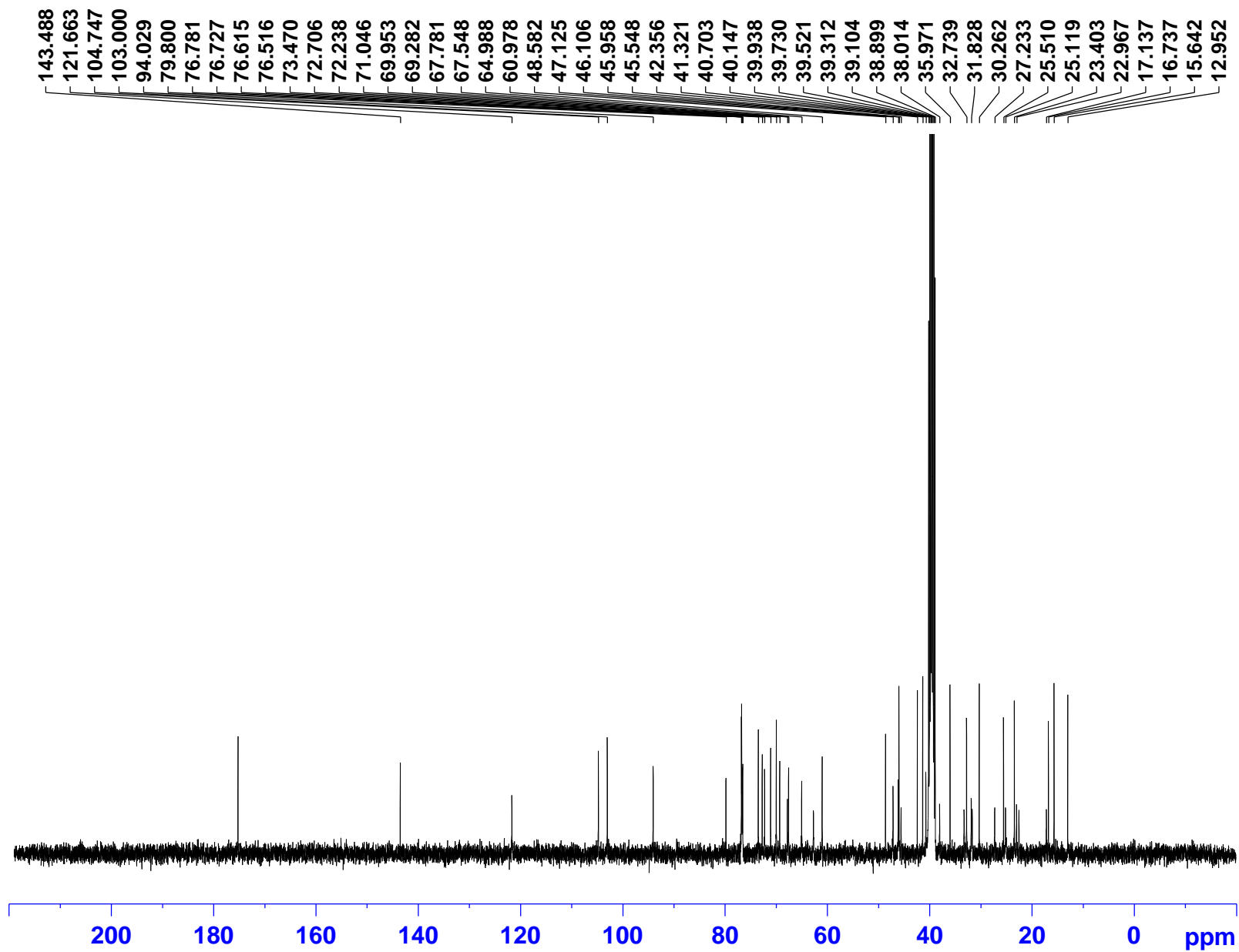

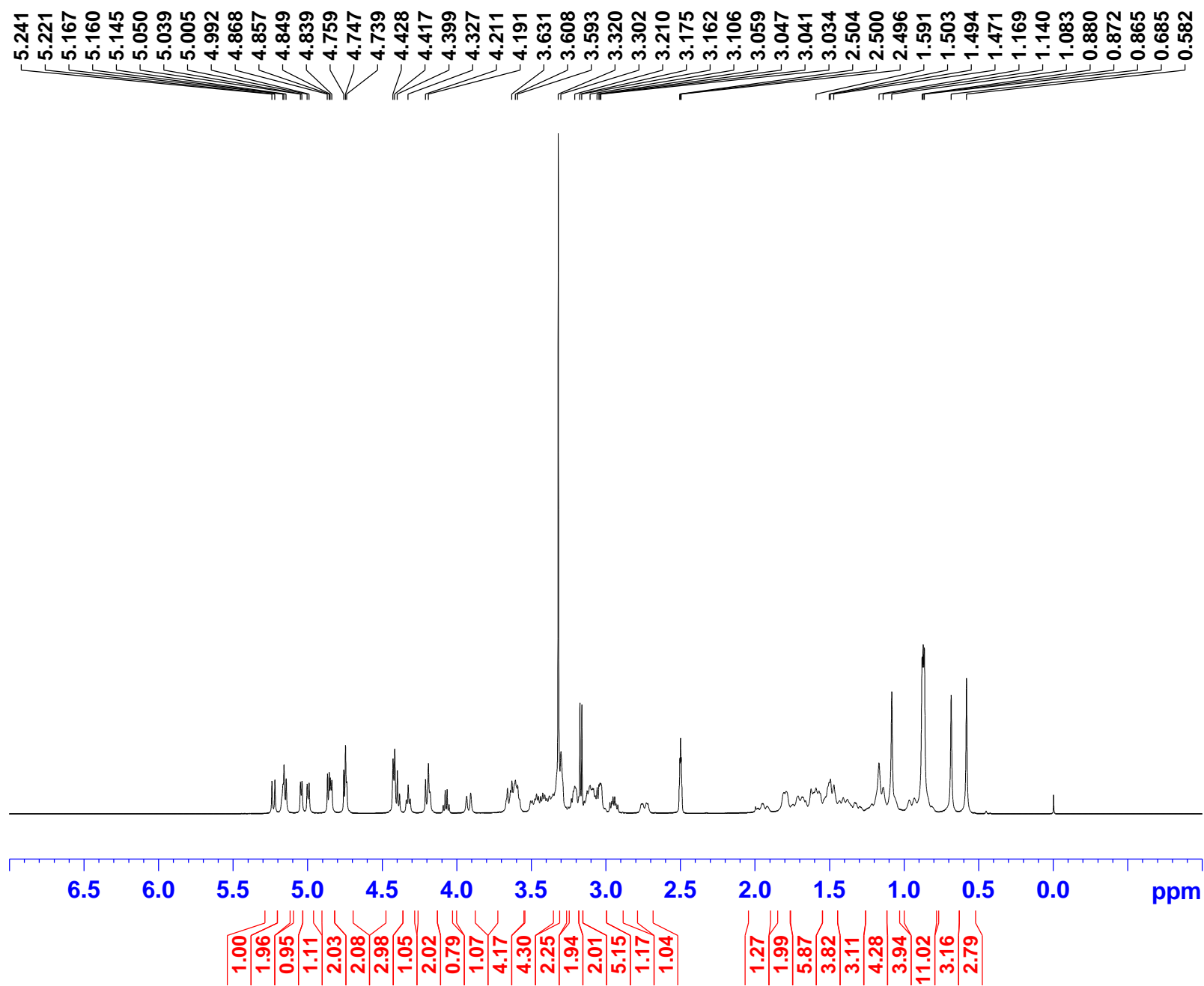

Supplement: Supplementary file 1 [file molecules-27-06236-s001.zip › The 1H-NMR of Akebia Saponin D- supplementary material.pdf]
